# Perioperative intraretinal fluid observed using optical coherence tomography in the epiretinal membrane

**DOI:** 10.1186/s12886-019-1289-5

**Published:** 2020-01-22

**Authors:** Jae Jung Lee, Yeon Ji Jo, Han Jo Kwon, Seung Min Lee, Sung Who Park, Ik Soo Byon, Ji Eun Lee

**Affiliations:** 1 Department of Ophthalmology, Pusan National University Hospital, 179 Gudeok-ro, Seo-gu, Busan, 49241 South Korea; 2 Department of Ophthalmology, School of Medicine, Pusan National University, Yangsan, South Korea; 3 Biomedical Research Institute, Pusan National University Hospital, Busan, South Korea; 4 Research Institute for Convergence of Biomedical Science and Technology, Pusan National University Yangsan Hospital, Yangsan, South Korea

**Keywords:** Epiretinal membrane, Intraretinal fluid, Macular edema, Optical coherence tomography, Vitrectomy

## Abstract

**Background:**

Postoperative intraretinal fluid (IRF) is reportedly associated with visual outcomes after epiretinal membrane (ERM) surgery. However, preoperative IRF is common, and persistent IRF would have different impact on visual function from postoperative newly developed IRF. Therefore, we aimed to investigate the incidence rate and clinical implications of perioperative IRF in ERM.

**Methods:**

Medical records of patients who underwent vitrectomy for idiopathic ERM between January 2014 and January 2017 were reviewed retrospectively. The incidence of IRF was analyzed using optical coherence tomography preoperatively and 1, 3, and 6 months postoperatively. On the basis of the presence or absence and the time of detection of IRF, patients were divided into three groups, namely preoperative IRF group, New IRF group, and IRF(−) group. Correlations of various parameters including age, sex, baseline visual acuity (VA), central subfield macular thickness, lens status, and surgical factors with IRF, along with the effect of IRF on VA, were evaluated.

**Results:**

This study included 155 eyes from 155 patients. Thirty-six (23.2%) and 49 (31.6%) eyes demonstrated preoperative and newly developed IRF, respectively. Seventy eyes (45.2%), which did not exhibit IRF during the study period, were assigned to the IRF(−) group. At baseline, the IRF(−) group showed a better VA than the other two groups. Postoperatively, VA improved significantly in all three groups. There was no difference in VA between the IRF(−) and new IRF groups at 6 months; however, the preoperative IRF group had significantly lower VA than the other two groups.

**Conclusion:**

IRF associated with ERM was frequently observed preoperatively and postoperatively, but it did not prevent postoperative vision improvement. Preoperative IRF was related to lower postoperative vision improvement.

## Background

The pathogenesis of epiretinal membrane (ERM) involves glial cell proliferation over the internal limiting membrane (ILM) of the retina. The prevalence of ERM is estimated to be between 7 and 11.8%, and it increases with an increase in age [1, 2]. The ERM frequently involves the macula, and pars plana vitrectomy (PPV) together with membrane peeling, with or without ILM peeling, is commonly performed for symptomatic ERM, resulting in favorable visual prognoses in most cases [3, 4].

Previous studies have reported the incidence of macular edema after PPV. Particularly for ERM, the incidence rates of macular edema after PPV varied between 13 and 64% [5–7]. Previously, macular edema was synonymous with macular thickening detected by ophthalmoscopic examinations. However, in the era of optical coherence tomography (OCT), macular edema can be differentiated from an increase in macular thickness by mechanical deformation; this term is reserved for macular thickening due to the presence of intraretinal fluid (IRF) or intraretinal cystoid changes shown by OCT. The presence of IRF was associated with a delay in postoperative visual improvement [5, 8]. Additionally, cystoid changes were also commonly observed before ERM surgery [5, 7]. In these cases, persistent preoperative IRF would introduce a bias in interpreting the impacts of postoperative IRF, which includes both persistent IRF and newly developed IRF.

The current study comprehensively investigated the clinical implications of perioperative IRF in idiopathic ERM by analyzing functional outcomes according to the presence of preoperative and postoperative IRF.

## Methods

Medical records of patients who underwent surgery, at Pusan National University Hospital, for idiopathic ERM between January 2014 and January 2017 were reviewed retrospectively. The institutional review board of Pusan National University Hospital approved the study protocol, which complied with the tenets of the Declaration of Helsinki.

Patients with secondary ERM (diabetic retinopathy, venous occlusion, uveitis, retinal detachment, trauma, etc.) were excluded from the study. Patients who had undergone intraocular surgery within 3 months before ERM surgery, had severe cataract before operation, had a postoperative follow-up period of less than 6 months, had undergone surgery performed by surgeons with less than 2 years of experience, or had an intraretinal space because of dehiscence of the retinal layers similar to retinoschisis were also excluded to prevent bias.

All patients underwent a complete ophthalmic examination, including slit-lamp examination with a 90D lens, fundus photography, and spectral domain OCT (Cirrus HD-OCT; Carl Zeiss Meditec, Inc., Dublin, CA, USA) or swept-source OCT (DRI-OCT1 Atlantis; Topcon Inc., Tokyo, Japan). Best-corrected visual acuity was measured before surgery and 1, 3, and 6 months postoperatively using the Snellen chart and was converted to the logarithm of minimum angle of resolution (logMAR) value for statistical analysis. Demographic data such as age, sex, lens status, and surgical factors (staining method, ILM peeling, concurrent cataract surgery, and duration of surgery) were reviewed.

All the surgeries were performed by two expert surgeons (JE Lee and SW Park). Three-port 25G PPV was performed using the Constellation (Alcon Laboratories, Inc., Fort Worth, TX, USA) with a RESIGHT 700 non-contact wide-angle viewing system (Carl Zeiss Meditec AG, Jena, Germany). Phacoemulsification was performed concurrently at the surgeon’s discretion. A clear corneal incision was made at the 12 O’clock position for phacoemulsification, following which vitrectomy was performed. Triamcinolone acetonide (TA) was applied to visualize the vitreous and ERM. The fibrous membrane was removed with intraocular forceps. The ILM was removed at the surgeon’s discretion after staining it with Brilliant Blue G (BBG). The sclerotomy site remained without a suture in most cases.

### OCT image analysis

OCT images were obtained at the posterior pole over a 6 × 6 mm^2^ or 12 × 9 mm^2^ area using the 3-D volume scan protocol for 128 (Cirrus HD-OCT) or 256 (DRI-OCT1 Atlantis) B-scans, each composed of 512 A-scans performed at baseline and 1, 3, and 6 months postoperatively. Mean central subfield macular thickness (CSMT) was calculated at each visit.

In all the B-scans, the 6 × 6 mm^2^ area centered at the fovea was reviewed, and if any IRF was detected, the patients were classified as IRF(+). IRF was defined as a distinct hypo-reflective space observed in the retinal layers. As mentioned above, the retinoschisis-type IRF was not regarded as a sign of macular edema, and such cases were excluded from the analysis. Two trained ophthalmologists (JJ Lee and YJ Jo) analyzed the OCT findings independently. For discrepancy assessment, the final decision was made on the basis of a consensus between the two physicians. Poor-quality images were excluded.

Furthermore, other qualitative assessments of OCT images, such as that of the presence of disorganization of inner nuclear layers (DRIL) and the integrity of external limiting membrane (ELM) and ellipsoid zone (EZ), were performed in 6 months OCT images, which was selected to avoid interpretation bias due to shadowing effects by the inner retinal pathology of early perioperative period. EZ integrity was graded as intact, attenuated, or disrupted on the basis of its appearance.

All statistical analyses were performed using IBM SPSS Statistics for Windows, version 21.0 (IBM Corp., Armonk, NY, USA). Comparisons were made among the three groups using Kruskal-Wallis test for continuous variables, and chi-squared test for categorical variables. For comparison between two groups, Mann-Whitney U test and Fisher’s exact test or chi-squared test were used. Comparisons between preoperative and postoperative values were made using the paired t-test or Wilcoxon signed-rank test. *p*-values < 0.05 were considered statistically significant.

## Results

### Perioperative IRF changes

This study included 155 eyes from 155 patients (50 men and 105 women) with a mean age of 65.2 years (range 43–82 years). Among 130 phakic eyes, 126 (96.9%) underwent concurrent phacoemulsification. Thirty-six eyes from 36 patients (23.2%) had preoperative IRF, as determined using OCT, and 119 eyes (76.8%) did not have IRF at baseline.

**Fig. 1:**
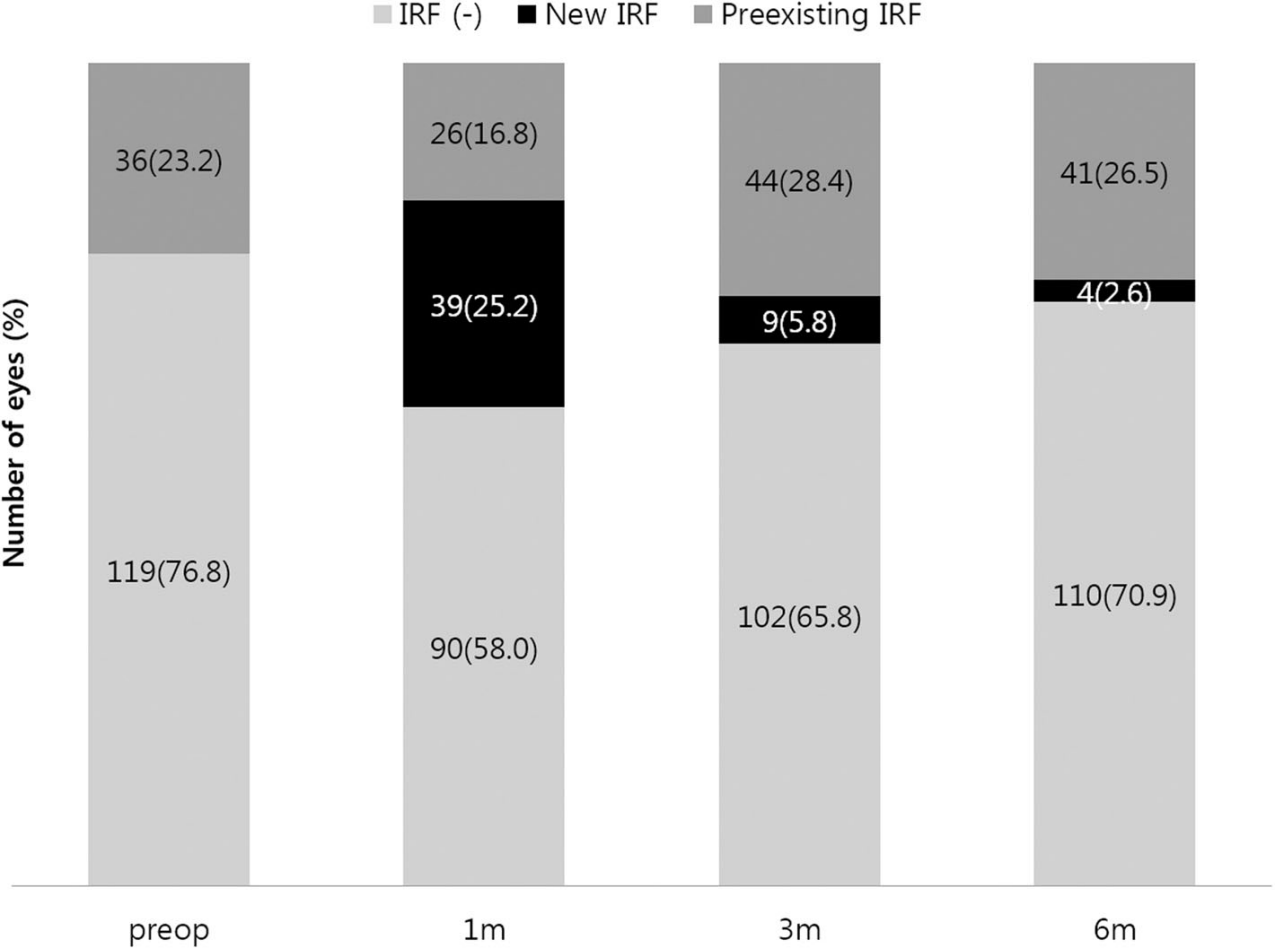
Proportions of eyes with and without intraretinal fluid (IRF) in the perioperative period. Newly developed IRF was detected 1, 3, and 6 months postoperatively; it decreased over time. The overall proportion of eyes with IRF also decreased

One month postoperatively, IRF was detected in 65 eyes (42.0%), including 39 eyes (25.2%) with newly developed IRF, and preexisting IRF was resolved in 10 eyes (6.5%). Three months postoperatively, IRF was detected in 53 eyes (34.2%) including nine eyes (5.8%) with newly developed IRF, and preexisting IRF was resolved in 21 eyes (13.5%). Six months postoperatively, OCT demonstrated IRF in 45 eyes (29.1%) including four eyes (2.6%) with newly developed IRF, and resolution of preexisting IRF was noted in 12 eyes (7.7%). The development of new IRF peaked 1 month postoperatively and then, decreased over time. The changes in the proportions of eyes with and without IRF, over time, are presented in Fig. 1. Overall, preoperative IRF persisted in 55.6% of the eyes (20 out of 36) and the newly developed IRF persisted in 51.0% of the eyes (25 out of 49), 6 months postoperatively.

Topical non-steroidal anti-inflammatory solution was administrated to seven patients, which led to the resolution of IRF in two of them. One patient with cystoid macular edema received intravitreal TA injection, which resulted in the resolution of IRF.

### Group analysis according to presence of IRF

**Fig. 2:**
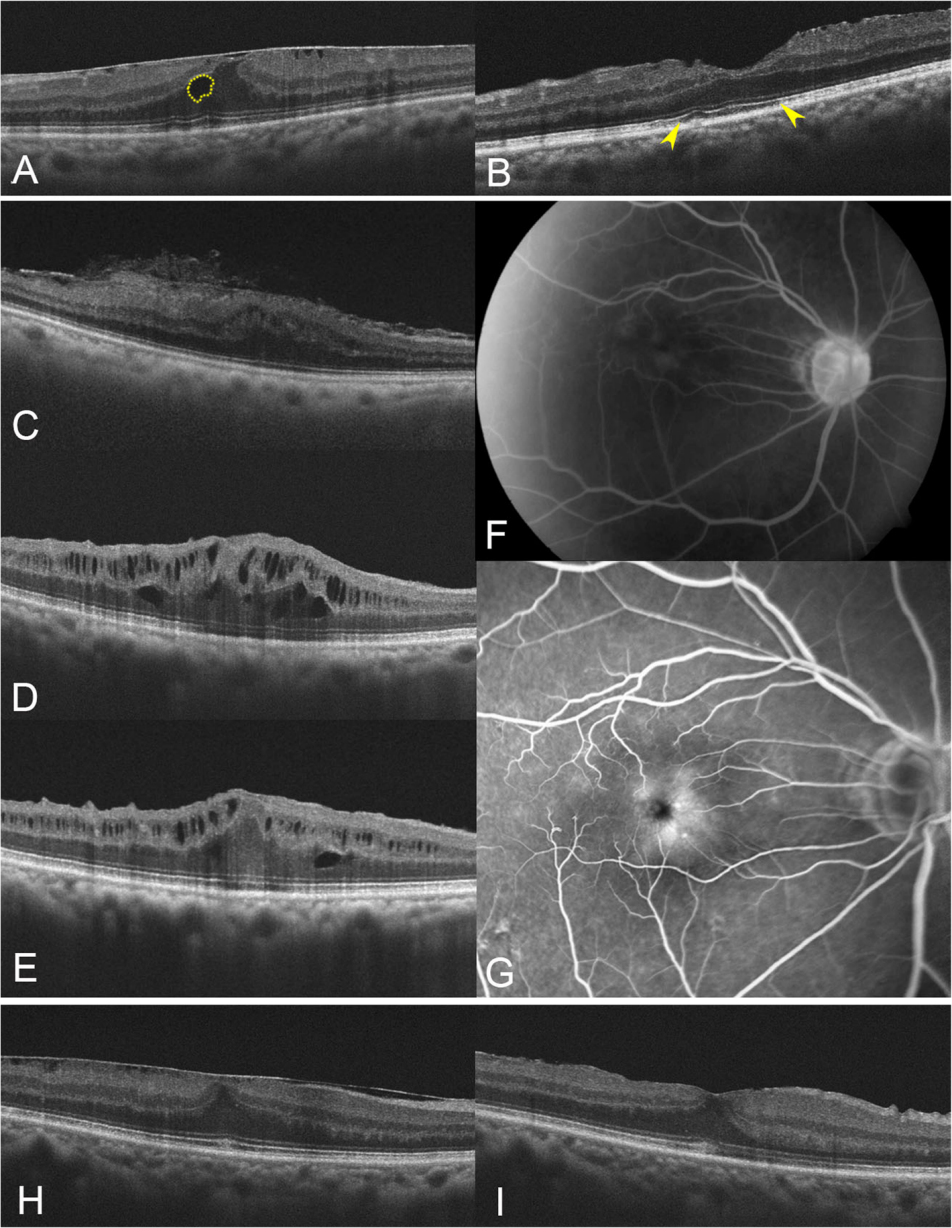
Representative cases of preoperative intraretinal fluid (IRF) (**a**, **b**), new IRF (**c**–**g**) and IRF(−) groups (**h**, **i**). **a** IRF (yellow dotted line) was observed in the baseline OCT image. **b** At 3 months postoperatively, IRF disappeared and disrupted ellipsoid zone (yellow arrowheads) was observed. **c**–**e** Serial changes in optical coherence tomography (OCT) findings in a patient without preoperative IRF. **c** No IRF was observed in the baseline OCT images. **d** Intraretinal fluid first appeared 1 month postoperatively and persisted up to (**e**) 1 year. Visual acuity improved from 20/200 at baseline to 20/125 at 1 year. **f** Fluorescein angiography, performed before surgery, demonstrated mild perifoveal microvascular leakage without disc leakage. **g** Prominent parafoveal microvascular leakage was noted in fluorescein angiography at 1 year postoperatively. **h** There was no IRF before surgery in patients of the IRF (−) group. **i** Until 6 months postoperatively, the OCT image showed no IRF

The patients were divided into three groups: preoperative IRF, new IRF, and IRF(−) groups. The new IRF group included the eyes that had newly developed IRF, as determined using OCT images, at any visit after surgery. Similarly, the IRF(−) group included the eyes that did not present with IRF at any visit during the study period (Fig. 2). These groups had 36 (23.2%), 49 (31.6%), and 70 eyes (45.2%), respectively.

The baseline characteristics were compared among the three groups. There was a statistically significant difference in age, baseline visual acuity, lens status, conduct of concurrent cataract surgery, and presence of DRIL. The mean age was significantly higher in the preoperative IRF group than in the new IRF and IRF(−) groups (68.1 ± 6.6, 64.6 ± 7.1, and 64.2 ± 8.2 years, respectively; *p* = 0.047, Kruskal-Wallis test). There were significant differences in the baseline visual acuity among the three groups (0.54 ± 0.27, 0.48 ± 0.28, and 0.38 ± 0.18 logMAR, respectively; *p* = 0.004). With regard to comparison between two groups, the IRF(−) group had significantly better vision than the preoperative IRF and new IRF groups (*p* = 0.001 and 0.026, respectively; Mann-Whitney U test); however, there was no difference between the preoperative IRF and new IRF groups (*p* = 0.241). Pseudophakia was noted in 30.6% of the patients in the preoperative IRF group and 20.0% of those in the IRF(−) group; however, it was not initially present in the new IRF group, and concurrent cataract surgery was performed in 95.9% of the patients in this group.

Regarding OCT findings at 6 months, DRIL was significantly more prevalent in the preoperative IRF group than in the new IRF and IRF(−) groups (19.4, 6.1, 2.9%, respectively; *p* = 0.008, chi-squared test). Integrity of EZ and ELM was not significantly different among the three groups. The other baseline characteristics, such as sex ratio, laterality, preoperative CSMT, and surgical and staining methods, were not significantly different among the three groups. All the characteristics are summarized in Table 1.

**Table 1:** Baseline characteristics of patients in the preoperative intraretinal fluid (IRF), new IRF, and IRF(−) groups

	Preoperative IRF(+)	New IRF	IRF(−)	*p*-value
Number of eyes (patients)	36 (36)	49 (49)	70 (70)	
Age (years)	68.1 ± 6.6	64.6 ± 7.1	64.2 ± 8.2	0.047^*^
Male/Female (patients)	7/29	18/31	25/45	0.171
Laterality (Right/Left)	18/18	22/27	33/37	0.874
Visual acuity				
median (Snellen)	0.32	0.32	0.45	0.008^*^
mean (logMAR)	0.54 ± 0.27	0.48 ± 0.28	0.38 ± 0.18	0.004^*^
CSMT (μm)	428.7 ± 96.9	441.1 ± 82.1	426.5 ± 51.9	0.550
Lens status (phakic/pseudophakic)	25/11	49/0	56/14	< 0.001^*^
Duration of surgery (minutes)	42.3 ± 12.8	43.6 ± 12.3	42.5 ± 17.0	0.701
Combined/PPV only	25/11	47/2	54/16	0.004^*^
No stain/TA/BBG	3/7/26	1/10/38	5/9/56	0.529
MP only/MP + ILMP	11/25	11/38	15/55	0.557
ILMP area (DD)	2.3 ± 1.7	2.6 ± 1.5	2.6 ± 1.5	0.895

*CSMT* central subfield macular thickness, *PPV* pars plana vitrectomy, *TA* triamcinolone acetonide, *BBG* Brilliant Blue G, *MP* membrane peeling, *ILMP* internal limiting membrane peeling, *DD* Disc diameter

*p < 0.05 was considered statistically significant

### Comparison of postoperative changes among three groups

**Fig. 3:**
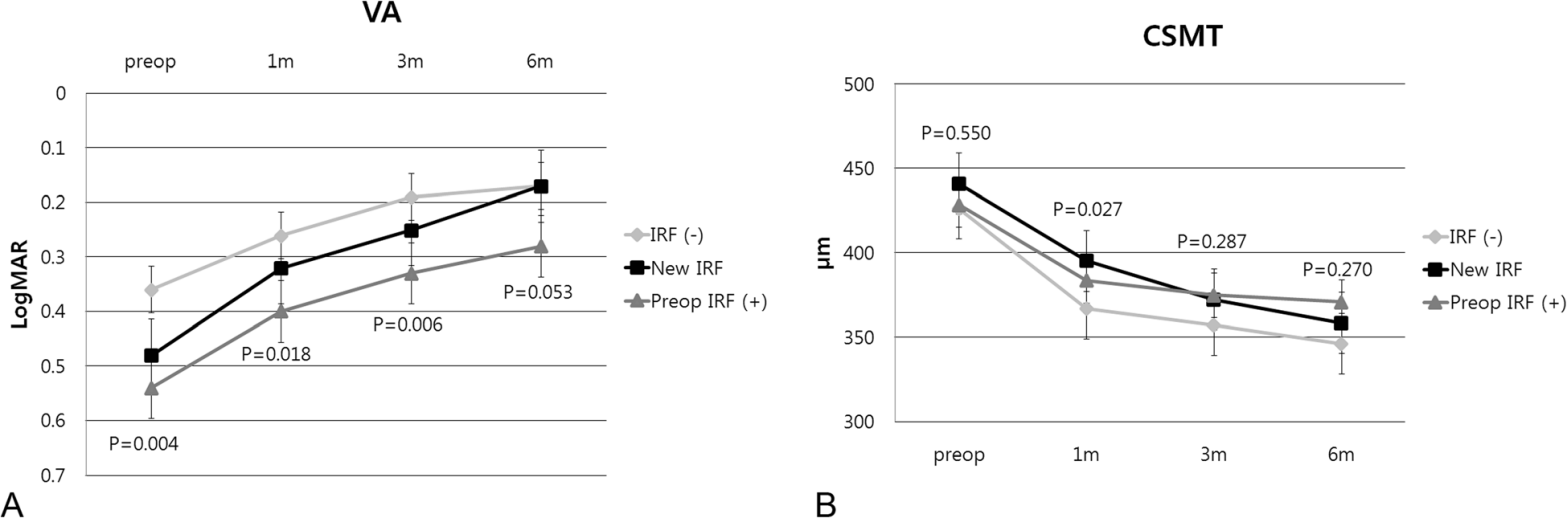
Changes in **a** visual acuity and **b** central subfield macular thickness in preoperative intraretinal fluid (IRF), new IRF, and IRF(−) groups after surgery for idiopathic epiretinal membrane. The bar indicates the interquartile range. (VA; visual acuity, CSMT; central subfield macular thickness, IRF; intraretinal fluid)

The visual acuity improved significantly from baseline to 6 month postoperatively in all of the preoperative IRF, new IRF and IRF(−) groups (0.28 ± 0.31, 0.17 ± 0.18, and 0.17 ± 0.19 logMAR, respectively, *p* < 0.001, Wilcoxon signed-rank test) and was not significantly different among the three groups (Fig. 3a). However, comparison between two groups showed changes in visual acuity, in detail. Visual improvement was prominent in the new IRF group, and no difference was found in visual acuity between the new IRF and IRF(−) groups (*p* = 0.760, Mann-Whitney U test) at 6 months. The preoperative IRF group had significantly lower visual acuity than the new IRF and IRF(−) groups (*p* = 0.049 and 0.021, respectively).

Preoperative IRF persisted in 55.6% of the eyes at 6 months postoperatively, and the visual improvement was compared within this group depending on whether IRF persisted (persistent IRF group vs resolved IRF group). Both sub-groups showed significant visual improvement (*p <* 0.001, Wilcoxon signed-rank test), and there was no significant difference in visual acuity at baseline and 6 months postoperatively between two sub-groups (*p* = 0.155 and 0.171, respectively, Mann-Whitney U test).

The mean CSMT decreased significantly after surgery in all groups. One month postoperatively, there was a significant difference in CSMT among the three groups (*p* = 0.027); CSMT decreased most prominently in the IRF(−) group. However, there were no differences between the groups at 3 and 6 months postoperatively (Fig. 3b).

Fluorescein angiography (FA) was performed in two patients with perioperative IRF, as determined using OCT. These two patients had phakic eyes and underwent vitrectomy combined with phacoemulsification. One patient exhibited preoperative IRF and macular edema, which worsened with an increase in IRF and subretinal fluid (SRF) after surgery. FA showed flower petal-pattern pooling and disc leakage in the late phase. Macular edema was resolved after the injection of intravitreal TA. The other patient exhibited no IRF at baseline and underwent FA, which revealed mild perifoveal microvascular leakage without disc leakage (Fig. 2). In this patient, IRF was newly developed at 1 month, and treated with topical non-steroidal anti-inflammatory drugs, which were not effective. Despite the presence of postoperative IRF, visual acuity improved from 20/200 at baseline to 20/125 at 6 months postoperatively. IRF persisted for 1 year after surgery, and FA images at this time showed more prominent parafoveal microvascular leakage in the late phase (Fig. 2f and g). TA was injected intravitreally; however, IRF persisted with minimal change in CSMT.

## Discussion

The IRF associated with ERM was frequently observed before and after surgery. We assessed the presence of IRF in the perioperative period and highlighted the various impacts on preoperative and postoperative visual outcomes. Generally, perioperative IRF was related to a lower preoperative visual acuity, but it did not prevent visual recovery after the surgery.

Although the literal definition of macular edema is the accumulation of fluid in the macula, it has been inconsistently defined in the literature. Traditionally, macular edema refers to thickening of the macula. However, the macula may be thickened by mechanical deformation in ERM, and the entirety of the thickening may not necessarily represent fluid accumulation. It has been reported that the CSMT could change even after 12 months of follow-up, but it does not reach the normal value [9]. Moreover, IRF observed using OCT has been associated with cystoid leakage detected using FA [10]. McDonald et al. [11] reported that the preoperative incidence rate of cystoid macular edema (CME) in idiopathic ERM was 71% (12 out of 17 eyes), as assessed using FA. However, FA findings did not always correspond to those observed using OCT, and the positioning of the fluid in the retinal layers is not obvious in FA [12, 13].

Accordingly, we used the term IRF instead of macular edema in the current study, as that is defined more specifically and consistently in the literature. OCT allows detailed imaging of the microstructures of the retinal layers and visualization of cystoid structures not seen using FA or an ophthalmoscope. Each cross-sectional image of 3-D volume scans was critically assessed to find even small IRFs.

The results from this study appear to be conflicting with those reported by previous studies. The incidence rate of postoperative IRF including persistent preoperative IRF was 29.1% at 6 months, and the incidence rate of new IRF was 41% (49 out of 119) in the eyes without preoperative IRF. Previous studies have reported widely varying postoperative IRF incidence rates, 13 to 64% [5–7], and new IRF incidence rates, 1 to 15.6% [5, 7, 8]. It is likely that the highest incidence rate [6] would represent thickening of the macula by both mechanical deformation and presence of fluid, as postoperative macular edema was assessed based on macular thickness measured by OCT and not based on the presence of IRF. The lowest incidence rate of about 1% indicated a proportion of delayed onset inner nuclear layer cystoid changes and not entirely that of the postoperative new IRF [8]. Frisina et al. [5] reported an incidence rate of about 10% for postoperative CME and new CME, as assessed by six radial scans (not a volume scan). Dolz-Marco et al. [7] compared the incidence rates of new IRF depending on whether combined phacoemulsification was performed or not, using macular cube scans, and reported the incidence to be 6.3% in the vitrectomy only group and 25% in the combined surgery group. In the current study, when patients were similarly divided into two subgroups, similar incidence rates, i.e., 6.9% among patients who underwent vitrectomy alone and 37.3% among those who underwent combined surgery, were observed. Overall, although the incidence rates of postoperative IRF appeared to show a wide variation in the literatures, they were comparable to ours considering the various factors associated.

Our study revealed that about one-fourth of patients had preoperative IRF and that these patients had significantly worse preoperative and postoperative visual acuity than other patients. Several previous studies reported the incidence rate of preoperative IRF to range from 13.6 to 40.6% using OCT [5, 7]. However, those reports did not analyze the impact of preoperative IRF on postoperative visual acuity. In the current study, visual acuity improved significantly after the surgery although preoperative IRF persisted in about half of the eyes for 6 months. The functional outcomes of the preoperative IRF group were worst among three groups despite the postoperative improvement. Our results were consistent with those of a recent prospective study that evaluated the risk factors of intraretinal cystoid changes after ERM surgery, which demonstrated that preoperative cystoid changes resulted in poorer postoperative visual outcomes [14].

The observations in the present study propose that preoperative factors are important in the development of IRF in ERM. This is supported by the differences in the baseline characteristics as well as the interesting findings of postoperative functional recovery. In general, visual function of the new IRF group was found to be intermediary to that of the other two groups. At baseline, visual acuity of the new IRF group was not different to that of the preoperative IRF group, but it was worse than that of the IRF(−) group. After surgery, the new IRF group demonstrated the most prominent visual improvement, which was not different from that of the IRF(−) group at 6 months. The preoperative IRF group had significantly worse visual acuity than the other two groups at 6 months postoperatively; however IRF was resolved in about half of them. Differences in the postoperative improvement between the new IRF and preoperative IRF groups suggests that preoperative IRF would indicate a more advanced stage with irreversible change, whereas new IRF group was at a less advanced stage with a relatively reversible damage. Furthermore, a more prevalent defect in DRIL indicated the irreversible structural change in the preoperative IRF group and supported our hypothesis.

The prominent improvement in the visual acuity of the new IRF group might be biased because it involved a higher rate of concurrent phacoemulsification. However, we excluded the cases with significant preoperative cataract, and visual acuity improved gradually during the 6-month follow-up period. The postoperative improvement in vision accordingly would be because of ERM surgery rather than because of cataract removal in the new IRF group. Furthermore, considering that more than 97% of the patients were pseudophakic postoperatively, the key findings of the current study, namely the worse outcomes of preoperative IRF group were still valid regardless of the functional effects of concurrent phacoemulsification.

Our results are somewhat contradictory to those of previous studies, which reported that postoperative IRF was related to worse functional outcomes. Sigler et al. [8] reported that new IRF was visually significant and led to a poor postoperative visual outcome in a small number of cases. However, their report was specifically about a finding of delayed onset IRF that had developed in the inner nuclear layer similar to the second case of FA in the present study and did not consider all cases of postoperative IRF. Another study did not differentiate the postoperative new IRF cases and the persistent preoperative IRF cases [5], which would be biased towards lowering the postoperative visual outcomes. Our novel findings of visual acuity changes related to perioperative IRF were obtained as we distinguished postoperative IRF into persistent IRF and new IRF to avoid the bias.

Several mechanisms have been suggested to underlie perioperative macular edema in idiopathic ERM. Glial cell proliferation on the retinal surface could cause damage to the vasculature by mechanical traction, which may result in vascular stasis and subsequent blood-retinal barrier (BRB) breakdown [15]. Postoperative inflammation is also one of the well-known causes. Frequent development of new IRF during the early postoperative period suggests that postoperative newly developed IRF would be associated with postoperative inflammation. Our study also demonstrated that new IRF developed most frequently at 1 month postoperatively. Release of inflammatory mediators triggered by surgery can break down the BRB and lead to an increase in vascular permeability, as observed in the case of pseudophakic CME [16]. This mechanism would be represented in this study by FA findings corresponding to the first case.

Macular edema is not an event influenced by a single factor, but it is rather a status of balance between many factors. Mechanical traction, venous stasis, and damaged BRB can cause vascular leakage; however, accumulation of IRF can be countered by physiological mechanisms that maintain the equilibrium of fluids. After ERM surgery, intraoperative damage and postoperative inflammation could put weights on the arm of IRF accumulation, whereas removal of membrane could attenuate the leakage caused by the preoperative factors. A combination of the factors noted above could tilt the balance towards either the development or resolution of IRF. Therefore, newly developed IRF represents a more advanced stage than the IRF(−) group does, but it still maintains a reversible status that could allow for a prominent improvement in vision after surgery. In contrast, preoperative IRF represents degenerative changes that exceed the issue of physiological balance even before surgery and would be a predictor of poorer functional outcomes than the other groups. These dynamic features of many participating factors were well-demonstrated in the two cases for which FA was performed in this study.

This study has some limitations. TA was used in some cases to enhance the visualization of ERM/IRF. TA is a steroid with anti-inflammatory properties, and it may affect the development of postoperative IRF. However, in our previous study, postoperative outcomes of ERM surgery were compared between two groups classified according to intravitreal or posterior subtenon TA injection at the end of the operation [17, 18]. No significant differences were found in terms of visual acuity and foveal thickness. In the present case series, a much smaller amount of TA was used just as an adjuvant for membrane peeling, and most of it was washed out immediately. Furthermore, intravitreal TA in a vitrectomized eye is known to be cleared very rapidly [19]. Consequently, the impact of TA on the results would be minimal.

The other limitations of the current study were its retrospective design, short follow-up period, the use of two types of OCT machines, and inclusion of a small cohort of patients in whom FA was performed. However, comprehensive analysis of perioperative IRF highlighted its various impacts on postoperative functional outcomes. Future studies are required to identify the specific OCT findings that may be useful for predicting the development of IRF and functional outcomes.

## Conclusion

In summary, perioperative IRF was commonly observed in ERM surgery, and the preoperative status would be an important factor involved in its development. IRF was resolved in many cases without any treatment other than ERM surgery, and visual acuity improved significantly regardless of IRF. Preoperative IRF had a clinical significance as it reflected a more advanced stage of ERM with an irreversible damage to the macula, and it is correlated to worse visual outcomes.

## Data Availability

The datasets used and/or analyzed during the current study are available from the corresponding author up on reasonable request.

## Abbreviations

BBG: Brilliant Blue G
BRB: Blood-retinal barrier
CME: Cystoid macular edema
CSMT: Central subfield macular thickness
DRIL: Disorganization of inner nuclear layers
ELM: External limiting membrane
ERM: Epiretinal membrane
EZ: Ellipsoid zone
FA: Fluorescein angiography
ILM: Internal limiting membrane
IRF: Intraretinal fluid
OCT: Optical coherence tomography
PPV: Pars plana vitrectomy
SRF: Subretinal fluid
TA: Triamcinolone acetonide
VA: Visual acuity
